# Tris[dimethyl (benzoyl­amido)­phosphato-κ^2^ *O*,*O*′](1,10-phenanthroline-κ^2^ *N*,*N*′)neodymium(III)

**DOI:** 10.1107/S1600536813003462

**Published:** 2013-02-09

**Authors:** Nataliia S. Kariaka, Victor A. Trush, Volodymyr V. Medviediev, Tetyana Yu. Sliva, Vladimir M. Amirkhanov

**Affiliations:** a Kyiv National Taras Shevchenko University, Department of Chemistry, Volodymyrska str. 64, 01601 Kyiv, Ukraine; b STC "Institute for Single Crystals", National Academy of Science of Ukraine, Lenina ave. 60, 61001 Khar’kov, Ukraine

## Abstract

In both independent mol­ecules of the title compound, [Nd(C_9_H_11_NO_4_P)_3_(C_12_H_8_N_2_)], the Nd^III^ atom is coordinated by six O atoms belonging to three phosphoryl ligands and two N atoms of 1,10-phenanthroline in a dodeca­hedral geometry. In the phosphoryl ligands, the benzene rings are twisted with respect to the planes of the *sp* ^2^-hybridized C atoms of the chelate rings by 12.1 (1)–24.7 (1)°.

## Related literature

For the phosphoryl ligand, see: Kirsanov (1954 ▶); Derkach *et al.* (1960 ▶); Mizrahi & Modro (1982 ▶). For the coordinating properties of carbacyl­amido­phosphates, see: Legendziewicz *et al.* (2000 ▶); Znovjyak *et al.* (2009 ▶). For related mol­ecules, see: Oczko *et al.* (2003 ▶); Malandrino *et al.* (1998 ▶). For the calculation of polyhedra of lanthanide anions, see: Porai-Koshits & Aslanov (1972 ▶).
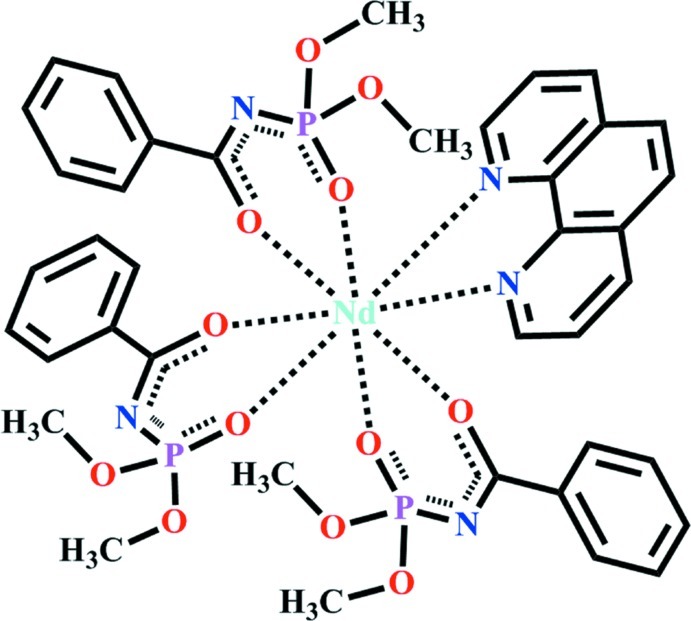


## Experimental

### 

#### Crystal data

[Nd(C_9_H_11_NO_4_P)_3_(C_12_H_8_N_2_)]
*M* *_r_* = 1008.92
Triclinic, 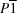

*a* = 13.0418 (4) Å
*b* = 17.8453 (7) Å
*c* = 21.8391 (9) Å
α = 106.915 (3)°
β = 104.977 (3)°
γ = 104.125 (3)°
*V* = 4408.5 (3) Å^3^
*Z* = 4
Mo *K*α radiation
μ = 1.35 mm^−1^
*T* = 293 K
0.3 × 0.2 × 0.1 mm

#### Data collection

Agilent Xcalibur Sapphire3 diffractometer
Absorption correction: multi-scan (*CrysAlis PRO*; Agilent, 2012 ▶) *T*_min_ = 0.735, *T*_max_ = 1.000
52874 measured reflections
25698 independent reflections
17831 reflections with *I* > 2σ(*I*)
*R*_int_ = 0.022

#### Refinement

*R*[*F* ^2^ > 2σ(*F* ^2^)] = 0.036
*wR*(*F* ^2^) = 0.089
*S* = 1.01
25698 reflections
1093 parameters
H-atom parameters constrained
Δρ_max_ = 0.86 e Å^−3^
Δρ_min_ = −0.96 e Å^−3^

### 

Data collection: *CrysAlis PRO* (Agilent, 2012 ▶); cell refinement: *CrysAlis PRO*; data reduction: *CrysAlis PRO*; program(s) used to solve structure: *SHELXS97* (Sheldrick, 2008 ▶); program(s) used to refine structure: *SHELXL97* (Sheldrick, 2008 ▶); molecular graphics: *SHELXTL* (Sheldrick, 2008 ▶); software used to prepare material for publication: *publCIF* (Westrip, 2010 ▶).

## Supplementary Material

Crystal structure: contains datablock(s) I, global. DOI: 10.1107/S1600536813003462/ng5315sup1.cif

Structure factors: contains datablock(s) I. DOI: 10.1107/S1600536813003462/ng5315Isup2.hkl

Additional supplementary materials:  crystallographic information; 3D view; checkCIF report

## Figures and Tables

**Fig. 1.**
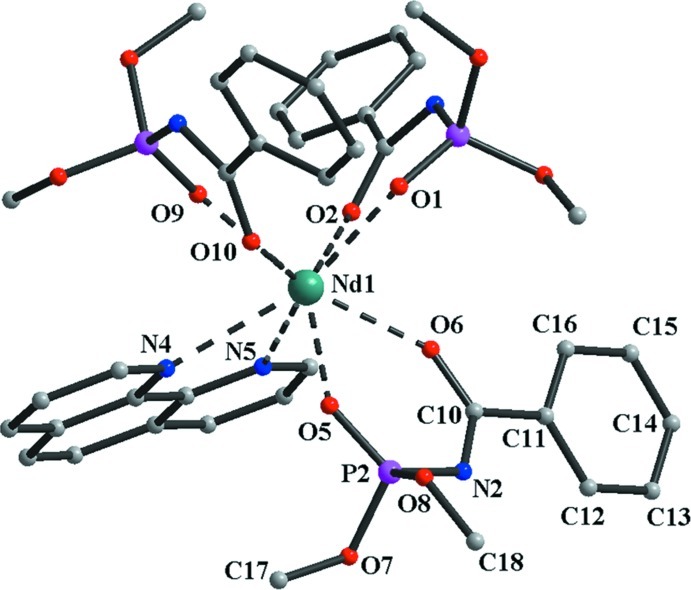
View of [NdL3Phen] molecule (Molecule 1) with atom numbering scheme. H atoms have been omitted.

**Fig. 2.**
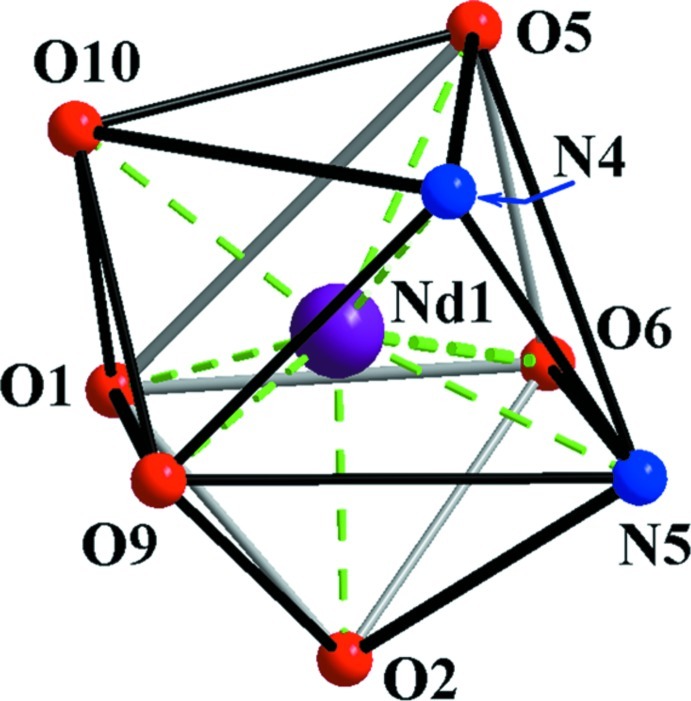
Polyhedron of Nd1 coordination center.

## supplementary crystallographic information

### Comment

Synthesis of luminescent lanthanide complexes (Oczko *et al.*, 2003, Legendziewicz, *et al.*, 2000) has been attracted a considerable interest because of their potential application, such as fluorescent labeling reagents and emitter materials in metal-organic light-emitting diodes. As a part of study of Ln(III) coordination compounds based on carbacylamidophosphates (CAPh), we have obtained the title compound [Nd*L*_3_Phen] (Fig. 1) (*L*^-^ is {C_6_H_5_CONPO(OCH_3_)_2_}^-^) and determined the crystal structure.

The complex has a molecular structure. The asymmetric unit of the [Nd*L*_3_Phen] crystal structure contains two crystallographically independent molecules. In each molecule Nd(III) has eight-coordinated environment formed by six O atoms of the carboxyl and phosphoryl groups of three bidentate-chelate ligands and two nitrogen atoms of 1,10- phenanthroline. According to the geometrical criteria proposed for determination of the form of eight-apical polyhedra (Porai-Koshits & Aslanov 1972) the resulting polyhedra of Nd^3+^ ions in both independent molecules of [Nd*L*_3_Phen] can be described as slightly distorted dodecahedron (Fig. 2).

The values of Nd—O distances are in a range of 2.3988 (16) - 2.4290 (16) Å for O atoms belonging to P=O groups and 2.3732 (17) - 2.4090 (14) Å for O atoms of carbonyl groups. The average P—O and C—O bond lengths increase in comparison to free ligand - (1.4818 (17) - 1.4890 (17) Å and 1.461 (4) Å for P—O groups of coordinated and free ligand and 1.244 (3) - 1.267 (3) Å and 1.219 (6) Å for C—O bond lengths respectively). The average P—N and C—N bond lengths decrease in comparison to free ligand (1.604 (2) - 1.612 (2) Å and 1.667 (5) Å for P—N groups of coordinated and free ligand and 1.304 (3) - 1.327 (3) Å and 1.393 (7) Å for C—N bond lengths respectively). Chelate 6-membered metal-cycles formed by oxygen ligands are characterized by deviations from mean-square planes, which do not exceed 0.37 Å with maximum one found for N(3) atom. Deviations of the Nd atoms from the mean planes defined by the other five atoms are in the range 0.08–0.49 Å. The bite angles around the central atom lay in range 70.89 (6) - 73.56 (6)° that is typical for lanthanide complexes with oxygen donor chelate ligands (Malandrino *et al.*, 1998; Znovjyak *et al.*, 2009). 1,10-phenanthroline is bidentate coordinated forming five-membered chelate cycle and the deviations from the plane plotted through atoms of chelate cycle do not exceed 0.11 Å. The crystal packing exhibits weak intermolecular H – O contacts between hydrogen atoms of 1,10- phenanthroline and oxygen atoms of phosphoryl and methyl groups of neighboring molecules, H – N contacts between hydrogen atoms of phenyl ring of CAPh-ligands and nitrogen atoms of chelating cycles and H – C contacts.

### Experimental

The synthesis of HL (Mizrahi & Modro, 1982) was carried out according to the method by Kirsanov (Kirsanov, 1954; Derkach *et al.*, 1960).

Nd(NO_3_)_3_.6H_2_O 0.4373 g (1 mmol) was dissolved in 2-propanol (15 ml). The solution was dehydrated by HC(OC_2_H_5_)_3_ (6 mmol) being heated to the boiling point and then cooled down. The resulting solution was added to solution of NaL 0.7535 g (3 mmol) in acetone (10 ml). Then the acetone solution (5 ml) of 1,10- phenanthroline 0.1802 g (1 mmol) was added. The mixture was heated to the boiling point and cooled down. After 15 minutes the precipitated NaNO_3_ was filtered and washed with 10 ml of cold i-PrOH. The resulting clear solution was left at ambient temperature for crystallization on air. The crystals were separated by filtration after \~ 48 h, washed with cool i-PrOH and finally dried on air. Yield: 0.80–0.86 g (80–85%). The single-crystal were obtained from acetone/2-propanol mixture of solvents.

### Refinement

All H atoms were positioned geometrically and refined using a riding model, with C—H = 0.93–0.96 Å and *U*ĩso~(H) = *xU*_eq_(C), where *x* = 1.5 for methyl H and 1.2 for all other H atoms. A rotating-group model was applied for the methyl groups.

### Crystal data

**Table:**

[Nd(C_9_H_11_NO_4_P)_3_(C_12_H_8_N_2_)]	*Z* = 4
*M**_r_* = 1008.92	*F*(000) = 2044
Triclinic, *P*1	*D*_x_ = 1.520 Mg m^−^^3^
*a* = 13.0418 (4) Å	Mo *K*α radiation, λ = 0.7107 Å
*b* = 17.8453 (7) Å	Cell parameters from 12027 reflections
*c* = 21.8391 (9) Å	θ = 3.0–32.4°
α = 106.915 (3)°	µ = 1.35 mm^−^^1^
β = 104.977 (3)°	*T* = 293 K
γ = 104.125 (3)°	Block, blue
*V* = 4408.5 (3) Å^3^	0.3 × 0.2 × 0.1 mm

### Data collection

**Table:**

Agilent Xcalibur Sapphire3 diffractometer	25698 independent reflections
Radiation source: Enhance (Mo) X-ray Source	17831 reflections with *I* > 2σ(*I*)
Graphite monochromator	*R*_int_ = 0.022
Detector resolution: 16.1827 pixels mm^-1^	θ_max_ = 30.0°, θ_min_ = 3.0°
ω scans	*h* = −18→18
Absorption correction: multi-scan (*CrysAlis PRO*; Agilent, 2012)	*k* = −25→24
*T*_min_ = 0.735, *T*_max_ = 1.000	*l* = −30→30
52874 measured reflections	

### Refinement

**Table:**

Refinement on *F*^2^	Primary atom site location: structure-invariant direct methods
Least-squares matrix: full	Secondary atom site location: difference Fourier map
*R*[*F*^2^ > 2σ(*F*^2^)] = 0.036	Hydrogen site location: inferred from neighbouring sites
*wR*(*F*^2^) = 0.089	H-atom parameters constrained
*S* = 1.01	*w* = 1/[σ^2^(*F*_o_^2^) + (0.0402*P*)^2^] where *P* = (*F*_o_^2^ + 2*F*_c_^2^)/3
25698 reflections	(Δ/σ)_max_ = 0.002
1093 parameters	Δρ_max_ = 0.86 e Å^−^^3^
0 restraints	Δρ_min_ = −0.96 e Å^−^^3^

### Special details

**Table:**

Experimental. Analysis found Nd 14.1% requires Nd 14.3%; IR (KBr pellet, cm-1) 1187 (s, PO) and 1520 (s, CO); 31P NMR (C6H6, p.p.m.) 45.1.
Geometry. All e.s.d.'s (except the e.s.d. in the dihedral angle between two l.s. planes) are estimated using the full covariance matrix. The cell e.s.d.'s are taken into account individually in the estimation of e.s.d.'s in distances, angles and torsion angles; correlations between e.s.d.'s in cell parameters are only used when they are defined by crystal symmetry. An approximate (isotropic) treatment of cell e.s.d.'s is used for estimating e.s.d.'s involving l.s. planes.
Refinement. Refinement of *F*^2^ against ALL reflections. The weighted *R*-factor *wR* and goodness of fit *S* are based on *F*^2^, conventional *R*-factors *R* are based on *F*, with *F* set to zero for negative *F*^2^. The threshold expression of *F*^2^ > σ(*F*^2^) is used only for calculating *R*-factors(gt) *etc*. and is not relevant to the choice of reflections for refinement. *R*-factors based on *F*^2^ are statistically about twice as large as those based on *F*, and *R*- factors based on ALL data will be even larger.

### Fractional atomic coordinates and isotropic or equivalent isotropic displacement parameters (Å^2^)

**Table:**

	*x*	*y*	*z*	*U*_iso_*/*U*_eq_	
Nd1	0.617917 (10)	0.295596 (7)	0.447837 (6)	0.03273 (4)	
Nd2	0.131636 (10)	0.293845 (7)	−0.049923 (6)	0.03388 (4)	
P1	0.49607 (5)	0.07434 (4)	0.36905 (3)	0.04061 (15)	
P2	0.58187 (5)	0.38150 (4)	0.31596 (4)	0.04062 (15)	
P3	0.56262 (6)	0.29911 (4)	0.60060 (4)	0.04168 (15)	
P4	0.07586 (6)	0.30703 (5)	0.10339 (4)	0.04714 (17)	
P5	0.00787 (6)	0.07152 (4)	−0.13032 (4)	0.04558 (16)	
P6	0.07716 (6)	0.36375 (5)	−0.19138 (4)	0.04486 (16)	
O1	0.48112 (14)	0.15713 (10)	0.38950 (9)	0.0451 (4)	
O2	0.71402 (14)	0.20039 (10)	0.46221 (10)	0.0493 (4)	
O3	0.50116 (18)	0.04425 (13)	0.29513 (10)	0.0627 (5)	
O4	0.38556 (15)	0.00442 (11)	0.35902 (10)	0.0550 (5)	
O5	0.56495 (17)	0.37367 (12)	0.37864 (10)	0.0561 (5)	
O6	0.66572 (19)	0.25316 (12)	0.34748 (10)	0.0619 (6)	
O7	0.65483 (16)	0.47342 (11)	0.32988 (11)	0.0615 (6)	
O8	0.46267 (17)	0.36677 (14)	0.26545 (11)	0.0636 (5)	
O9	0.64153 (14)	0.29885 (11)	0.56229 (9)	0.0473 (4)	
O10	0.44834 (13)	0.30651 (10)	0.46812 (9)	0.0417 (4)	
O11	0.57924 (16)	0.24521 (13)	0.64584 (10)	0.0546 (5)	
O12	0.59086 (18)	0.38675 (12)	0.65684 (11)	0.0627 (5)	
O13	0.15669 (15)	0.30351 (12)	0.06661 (9)	0.0522 (5)	
O14	−0.04023 (14)	0.30238 (11)	−0.03248 (9)	0.0438 (4)	
O15	0.09593 (19)	0.26448 (16)	0.15697 (11)	0.0723 (6)	
O16	0.10637 (18)	0.39980 (14)	0.15140 (11)	0.0728 (6)	
O17	−0.00489 (14)	0.15508 (11)	−0.11238 (10)	0.0513 (4)	
O18	0.22431 (15)	0.19880 (10)	−0.03254 (10)	0.0505 (4)	
O19	0.01081 (19)	0.03674 (13)	−0.20443 (11)	0.0669 (6)	
O20	−0.10225 (16)	0.00374 (12)	−0.13756 (11)	0.0636 (5)	
O21	0.07564 (16)	0.36750 (11)	−0.12301 (9)	0.0505 (5)	
O22	0.1843 (2)	0.24942 (13)	−0.14800 (11)	0.0673 (6)	
O23	0.13856 (19)	0.45428 (13)	−0.18615 (12)	0.0683 (6)	
O24	−0.04399 (18)	0.34463 (16)	−0.24230 (11)	0.0733 (6)	
N1	0.60160 (18)	0.06224 (12)	0.41766 (12)	0.0476 (5)	
N2	0.64305 (19)	0.32649 (14)	0.27692 (11)	0.0468 (5)	
N3	0.42936 (17)	0.26788 (14)	0.55794 (12)	0.0481 (5)	
N4	0.69678 (16)	0.45475 (11)	0.53438 (10)	0.0379 (4)	
N5	0.83932 (16)	0.37799 (12)	0.49587 (11)	0.0405 (5)	
N6	−0.05765 (18)	0.26722 (15)	0.05877 (12)	0.0510 (6)	
N7	0.11368 (19)	0.06011 (13)	−0.08186 (12)	0.0543 (6)	
N8	0.1323 (2)	0.30280 (15)	−0.23004 (11)	0.0521 (6)	
N9	0.35314 (17)	0.37867 (12)	0.00072 (12)	0.0454 (5)	
N10	0.20596 (17)	0.45473 (12)	0.03140 (11)	0.0436 (5)	
C1	0.69496 (19)	0.12614 (14)	0.45706 (12)	0.0340 (5)	
C2	0.7893 (2)	0.11032 (15)	0.50094 (12)	0.0376 (5)	
C3	0.7979 (2)	0.03164 (17)	0.48621 (16)	0.0550 (7)	
H3	0.7429	−0.0134	0.4486	0.066*	
C4	0.8883 (3)	0.0197 (2)	0.5275 (2)	0.0704 (9)	
H4	0.8940	−0.0332	0.5168	0.085*	
C5	0.9681 (3)	0.0841 (3)	0.58316 (19)	0.0721 (10)	
H5	1.0282	0.0754	0.6104	0.087*	
C6	0.9603 (3)	0.1626 (2)	0.59936 (16)	0.0689 (9)	
H6	1.0146	0.2068	0.6379	0.083*	
C7	0.8715 (2)	0.17554 (18)	0.55812 (14)	0.0507 (6)	
H7	0.8670	0.2288	0.5690	0.061*	
C8	0.4256 (4)	0.0552 (3)	0.24105 (18)	0.0971 (13)	
H8A	0.4312	0.0259	0.1983	0.146*	
H8B	0.4450	0.1135	0.2490	0.146*	
H8C	0.3495	0.0335	0.2394	0.146*	
C9	0.3389 (3)	0.0148 (2)	0.41203 (19)	0.0767 (10)	
H9A	0.2688	−0.0303	0.3969	0.115*	
H9B	0.3255	0.0668	0.4227	0.115*	
H9C	0.3909	0.0147	0.4521	0.115*	
C10	0.67470 (19)	0.27015 (14)	0.29668 (12)	0.0365 (5)	
C11	0.73026 (19)	0.22081 (15)	0.25593 (12)	0.0381 (5)	
C12	0.7661 (2)	0.24398 (17)	0.20763 (13)	0.0445 (6)	
H12	0.7530	0.2891	0.1982	0.053*	
C13	0.8219 (2)	0.1993 (2)	0.17321 (14)	0.0574 (8)	
H13	0.8472	0.2151	0.1413	0.069*	
C14	0.8394 (2)	0.1318 (2)	0.18644 (15)	0.0576 (8)	
H14	0.8770	0.1023	0.1637	0.069*	
C15	0.8018 (3)	0.10819 (19)	0.23284 (16)	0.0592 (8)	
H15	0.8120	0.0615	0.2406	0.071*	
C16	0.7483 (2)	0.15297 (17)	0.26882 (14)	0.0501 (7)	
H16	0.7248	0.1373	0.3014	0.060*	
C17	0.6256 (3)	0.54325 (19)	0.3659 (2)	0.1005 (14)	
H17A	0.5497	0.5369	0.3408	0.151*	
H17B	0.6769	0.5946	0.3698	0.151*	
H17C	0.6311	0.5441	0.4108	0.151*	
C18	0.4477 (4)	0.3802 (3)	0.2043 (2)	0.1243 (17)	
H18A	0.3695	0.3538	0.1752	0.186*	
H18B	0.4931	0.3570	0.1815	0.186*	
H18C	0.4702	0.4391	0.2141	0.186*	
C19	0.39075 (19)	0.27265 (14)	0.49775 (13)	0.0370 (5)	
C20	0.2656 (2)	0.23339 (14)	0.46081 (14)	0.0407 (6)	
C21	0.1920 (2)	0.21578 (17)	0.49471 (16)	0.0513 (7)	
H21	0.2209	0.2290	0.5417	0.062*	
C22	0.0768 (2)	0.1790 (2)	0.46014 (19)	0.0632 (8)	
H22	0.0287	0.1677	0.4837	0.076*	
C23	0.0339 (3)	0.1593 (2)	0.3917 (2)	0.0782 (10)	
H23	−0.0438	0.1346	0.3684	0.094*	
C24	0.1045 (3)	0.1755 (2)	0.3565 (2)	0.0878 (12)	
H24	0.0746	0.1613	0.3095	0.105*	
C25	0.2206 (3)	0.2132 (2)	0.39115 (17)	0.0645 (8)	
H25	0.2682	0.2249	0.3673	0.077*	
C26	0.5705 (3)	0.1602 (2)	0.6125 (2)	0.0832 (11)	
H26A	0.4946	0.1284	0.5810	0.125*	
H26B	0.5890	0.1364	0.6463	0.125*	
H26C	0.6220	0.1590	0.5881	0.125*	
C27	0.7027 (3)	0.4328 (2)	0.70525 (17)	0.0877 (11)	
H27A	0.7552	0.4364	0.6816	0.132*	
H27B	0.7213	0.4050	0.7359	0.132*	
H27C	0.7070	0.4879	0.7309	0.132*	
C28	0.9103 (2)	0.34192 (16)	0.47721 (16)	0.0518 (7)	
H28	0.8799	0.2904	0.4410	0.062*	
C29	1.0268 (2)	0.37663 (19)	0.50861 (19)	0.0603 (8)	
H29	1.0728	0.3493	0.4932	0.072*	
C30	1.0727 (2)	0.45103 (19)	0.56216 (19)	0.0628 (9)	
H30	1.1508	0.4748	0.5840	0.075*	
C31	1.0027 (2)	0.49242 (16)	0.58481 (15)	0.0488 (6)	
C32	0.88484 (19)	0.45315 (14)	0.54909 (13)	0.0388 (5)	
C33	1.0441 (2)	0.57186 (18)	0.64032 (17)	0.0617 (8)	
H33	1.1216	0.5975	0.6642	0.074*	
C34	0.9747 (3)	0.60949 (18)	0.65829 (16)	0.0620 (8)	
H34	1.0044	0.6612	0.6942	0.074*	
C35	0.8547 (2)	0.57211 (16)	0.62352 (14)	0.0490 (6)	
C36	0.8097 (2)	0.49393 (14)	0.56918 (13)	0.0380 (5)	
C37	0.7775 (3)	0.60919 (17)	0.64110 (16)	0.0607 (8)	
H37	0.8037	0.6604	0.6773	0.073*	
C38	0.6650 (3)	0.57046 (18)	0.60538 (16)	0.0593 (8)	
H38	0.6136	0.5953	0.6158	0.071*	
C39	0.6282 (2)	0.49274 (16)	0.55277 (14)	0.0480 (6)	
H39	0.5508	0.4660	0.5292	0.058*	
C40	−0.0976 (2)	0.26976 (15)	−0.00210 (13)	0.0404 (5)	
C41	−0.2227 (2)	0.23077 (15)	−0.03819 (15)	0.0459 (6)	
C42	−0.2954 (2)	0.22200 (19)	−0.00240 (18)	0.0612 (8)	
H42	−0.2661	0.2409	0.0451	0.073*	
C43	−0.4111 (3)	0.1855 (2)	−0.0366 (2)	0.0774 (11)	
H43	−0.4592	0.1799	−0.0120	0.093*	
C44	−0.4542 (3)	0.1579 (2)	−0.1056 (3)	0.0912 (13)	
H44	−0.5321	0.1339	−0.1283	0.109*	
C45	−0.3838 (3)	0.1648 (3)	−0.1427 (2)	0.0957 (13)	
H45	−0.4138	0.1447	−0.1903	0.115*	
C46	−0.2674 (3)	0.2022 (2)	−0.10834 (18)	0.0699 (9)	
H46	−0.2196	0.2078	−0.1330	0.084*	
C47	0.1010 (4)	0.1837 (3)	0.1365 (3)	0.125 (2)	
H47A	0.0313	0.1458	0.1006	0.187*	
H47B	0.1128	0.1666	0.1748	0.187*	
H47C	0.1625	0.1835	0.1200	0.187*	
C48	0.0262 (3)	0.4297 (2)	0.1747 (2)	0.1070 (15)	
H48A	−0.0391	0.4182	0.1361	0.161*	
H48B	0.0595	0.4888	0.2004	0.161*	
H48C	0.0040	0.4022	0.2035	0.161*	
C49	0.2056 (2)	0.12378 (14)	−0.03989 (12)	0.0367 (5)	
C50	0.2982 (2)	0.10667 (15)	0.00392 (13)	0.0396 (5)	
C51	0.3059 (3)	0.02798 (19)	−0.00990 (17)	0.0665 (8)	
H51	0.2513	−0.0172	−0.0476	0.080*	
C52	0.3954 (3)	0.0163 (2)	0.0326 (2)	0.0809 (11)	
H52	0.4008	−0.0366	0.0226	0.097*	
C53	0.4739 (3)	0.0804 (3)	0.0880 (2)	0.0775 (10)	
H53	0.5338	0.0719	0.1157	0.093*	
C54	0.4659 (3)	0.1580 (2)	0.10371 (18)	0.0763 (10)	
H54	0.5190	0.2020	0.1428	0.092*	
C55	0.3787 (2)	0.17106 (19)	0.06154 (15)	0.0570 (7)	
H55	0.3743	0.2243	0.0723	0.068*	
C56	−0.0655 (4)	0.0440 (3)	−0.26010 (18)	0.1022 (14)	
H56A	−0.0614	0.0112	−0.3023	0.153*	
H56B	−0.0457	0.1014	−0.2553	0.153*	
H56C	−0.1411	0.0243	−0.2604	0.153*	
C57	−0.1442 (3)	0.0161 (2)	−0.0821 (2)	0.0926 (12)	
H57A	−0.2146	−0.0279	−0.0955	0.139*	
H57B	−0.1557	0.0688	−0.0707	0.139*	
H57C	−0.0903	0.0157	−0.0429	0.139*	
C58	0.1793 (2)	0.25685 (16)	−0.20392 (13)	0.0407 (6)	
C59	0.2349 (2)	0.20720 (15)	−0.24419 (12)	0.0400 (5)	
C60	0.2657 (2)	0.22763 (18)	−0.29488 (13)	0.0500 (7)	
H60	0.2476	0.2700	−0.3068	0.060*	
C61	0.3239 (2)	0.1842 (2)	−0.32791 (15)	0.0615 (8)	
H61	0.3472	0.1990	−0.3609	0.074*	
C62	0.3469 (3)	0.1203 (2)	−0.31213 (16)	0.0655 (9)	
H62	0.3857	0.0916	−0.3345	0.079*	
C63	0.3135 (3)	0.09776 (19)	−0.26378 (17)	0.0654 (8)	
H63	0.3272	0.0529	−0.2543	0.078*	
C64	0.2587 (2)	0.14274 (18)	−0.22884 (15)	0.0562 (7)	
H64	0.2381	0.1288	−0.1948	0.067*	
C65	0.1555 (4)	0.4686 (3)	−0.2446 (2)	0.1210 (17)	
H65A	0.0836	0.4499	−0.2806	0.181*	
H65B	0.2014	0.4383	−0.2601	0.181*	
H65C	0.1927	0.5271	−0.2324	0.181*	
C66	−0.1299 (3)	0.2653 (3)	−0.2587 (2)	0.1158 (16)	
H66A	−0.1822	0.2468	−0.3046	0.174*	
H66B	−0.1697	0.2721	−0.2273	0.174*	
H66C	−0.0946	0.2247	−0.2552	0.174*	
C67	0.4255 (2)	0.34355 (17)	−0.01629 (17)	0.0571 (8)	
H67	0.3969	0.2913	−0.0516	0.068*	
C68	0.5426 (3)	0.3810 (2)	0.0161 (2)	0.0690 (9)	
H68	0.5904	0.3547	0.0019	0.083*	
C69	0.5851 (2)	0.4557 (2)	0.0682 (2)	0.0720 (10)	
H69	0.6629	0.4811	0.0907	0.086*	
C70	0.5126 (2)	0.49542 (18)	0.08852 (16)	0.0572 (7)	
C71	0.3956 (2)	0.45465 (15)	0.05225 (14)	0.0449 (6)	
C72	0.5508 (3)	0.5757 (2)	0.14362 (19)	0.0777 (10)	
H72	0.6277	0.6022	0.1690	0.093*	
C73	0.4783 (3)	0.6129 (2)	0.15896 (19)	0.0767 (10)	
H73	0.5056	0.6647	0.1947	0.092*	
C74	0.3599 (3)	0.57442 (17)	0.12140 (15)	0.0554 (7)	
C75	0.3180 (2)	0.49520 (14)	0.06853 (13)	0.0417 (6)	
C76	0.2810 (3)	0.61253 (18)	0.13343 (17)	0.0688 (9)	
H76	0.3054	0.6653	0.1675	0.083*	
C77	0.1700 (3)	0.57299 (19)	0.09574 (17)	0.0681 (9)	
H77	0.1174	0.5982	0.1032	0.082*	
C78	0.1357 (3)	0.49342 (17)	0.04531 (15)	0.0591 (8)	
H78	0.0588	0.4662	0.0201	0.071*	

### Atomic displacement parameters (Å^2^)

**Table:**

	*U*^11^	*U*^22^	*U*^33^	*U*^12^	*U*^13^	*U*^23^
Nd1	0.03239 (7)	0.02905 (7)	0.03956 (8)	0.01171 (5)	0.01436 (6)	0.01453 (6)
Nd2	0.03584 (7)	0.03235 (7)	0.03512 (8)	0.01356 (5)	0.01268 (6)	0.01353 (6)
P1	0.0384 (3)	0.0307 (3)	0.0429 (4)	0.0078 (3)	0.0088 (3)	0.0087 (3)
P2	0.0395 (3)	0.0461 (4)	0.0442 (4)	0.0218 (3)	0.0154 (3)	0.0219 (3)
P3	0.0396 (3)	0.0483 (4)	0.0411 (4)	0.0164 (3)	0.0127 (3)	0.0226 (3)
P4	0.0478 (4)	0.0615 (5)	0.0392 (4)	0.0248 (4)	0.0158 (3)	0.0235 (4)
P5	0.0421 (3)	0.0352 (3)	0.0442 (4)	0.0085 (3)	0.0049 (3)	0.0071 (3)
P6	0.0464 (4)	0.0568 (4)	0.0400 (4)	0.0275 (3)	0.0144 (3)	0.0229 (3)
O1	0.0363 (9)	0.0338 (9)	0.0572 (12)	0.0109 (7)	0.0088 (8)	0.0144 (8)
O2	0.0385 (9)	0.0332 (9)	0.0744 (13)	0.0131 (8)	0.0126 (9)	0.0239 (9)
O3	0.0707 (14)	0.0609 (13)	0.0465 (12)	0.0186 (11)	0.0190 (11)	0.0116 (10)
O4	0.0467 (10)	0.0413 (10)	0.0642 (13)	0.0040 (8)	0.0139 (10)	0.0167 (10)
O5	0.0804 (14)	0.0680 (12)	0.0555 (12)	0.0540 (11)	0.0392 (11)	0.0360 (10)
O6	0.0987 (16)	0.0619 (12)	0.0672 (14)	0.0516 (12)	0.0570 (13)	0.0385 (11)
O7	0.0557 (12)	0.0461 (11)	0.0925 (17)	0.0241 (10)	0.0343 (12)	0.0271 (11)
O8	0.0521 (11)	0.0821 (15)	0.0576 (13)	0.0353 (11)	0.0092 (11)	0.0268 (12)
O9	0.0391 (9)	0.0617 (11)	0.0468 (11)	0.0189 (9)	0.0146 (8)	0.0278 (9)
O10	0.0392 (9)	0.0471 (10)	0.0496 (11)	0.0204 (8)	0.0195 (8)	0.0252 (9)
O11	0.0515 (11)	0.0669 (13)	0.0591 (13)	0.0250 (10)	0.0192 (10)	0.0395 (11)
O12	0.0666 (13)	0.0568 (13)	0.0538 (13)	0.0241 (11)	0.0109 (11)	0.0129 (11)
O13	0.0472 (10)	0.0718 (13)	0.0453 (11)	0.0249 (10)	0.0166 (9)	0.0290 (10)
O14	0.0411 (9)	0.0520 (10)	0.0472 (11)	0.0205 (8)	0.0190 (8)	0.0244 (9)
O15	0.0791 (15)	0.1132 (19)	0.0650 (14)	0.0552 (14)	0.0380 (13)	0.0603 (14)
O16	0.0632 (13)	0.0711 (15)	0.0654 (15)	0.0248 (12)	0.0149 (12)	0.0056 (12)
O17	0.0408 (9)	0.0398 (10)	0.0570 (12)	0.0133 (8)	0.0023 (9)	0.0093 (9)
O18	0.0436 (10)	0.0354 (9)	0.0632 (13)	0.0120 (8)	0.0048 (9)	0.0197 (9)
O19	0.0740 (14)	0.0623 (13)	0.0489 (13)	0.0212 (11)	0.0149 (12)	0.0074 (11)
O20	0.0517 (12)	0.0500 (12)	0.0704 (14)	0.0023 (9)	0.0118 (11)	0.0183 (11)
O21	0.0695 (12)	0.0552 (11)	0.0472 (11)	0.0399 (10)	0.0268 (10)	0.0270 (9)
O22	0.1136 (18)	0.0769 (14)	0.0640 (14)	0.0676 (14)	0.0610 (14)	0.0443 (12)
O23	0.0879 (16)	0.0633 (13)	0.0795 (16)	0.0357 (12)	0.0416 (14)	0.0441 (13)
O24	0.0538 (12)	0.0974 (18)	0.0611 (14)	0.0368 (13)	0.0064 (11)	0.0231 (13)
N1	0.0472 (12)	0.0313 (11)	0.0537 (14)	0.0136 (9)	0.0070 (11)	0.0108 (10)
N2	0.0548 (13)	0.0554 (14)	0.0453 (13)	0.0300 (11)	0.0249 (11)	0.0244 (11)
N3	0.0377 (11)	0.0627 (14)	0.0508 (14)	0.0172 (10)	0.0171 (11)	0.0297 (12)
N4	0.0367 (10)	0.0338 (10)	0.0445 (12)	0.0142 (9)	0.0135 (10)	0.0156 (9)
N5	0.0365 (10)	0.0307 (10)	0.0572 (14)	0.0118 (9)	0.0211 (10)	0.0166 (10)
N6	0.0443 (12)	0.0665 (15)	0.0460 (13)	0.0180 (11)	0.0168 (11)	0.0266 (12)
N7	0.0505 (13)	0.0361 (12)	0.0589 (15)	0.0139 (10)	0.0004 (12)	0.0113 (11)
N8	0.0602 (14)	0.0699 (16)	0.0367 (12)	0.0370 (13)	0.0181 (11)	0.0219 (12)
N9	0.0408 (11)	0.0358 (11)	0.0611 (15)	0.0125 (9)	0.0202 (11)	0.0189 (11)
N10	0.0430 (11)	0.0372 (11)	0.0442 (13)	0.0172 (10)	0.0069 (10)	0.0116 (10)
C1	0.0382 (12)	0.0323 (12)	0.0360 (13)	0.0167 (10)	0.0161 (11)	0.0126 (10)
C2	0.0408 (13)	0.0409 (13)	0.0370 (14)	0.0189 (11)	0.0169 (11)	0.0162 (11)
C3	0.0581 (17)	0.0458 (15)	0.0623 (19)	0.0271 (14)	0.0162 (15)	0.0189 (14)
C4	0.073 (2)	0.072 (2)	0.093 (3)	0.0485 (19)	0.031 (2)	0.046 (2)
C5	0.062 (2)	0.111 (3)	0.069 (2)	0.048 (2)	0.0243 (19)	0.053 (2)
C6	0.0562 (18)	0.096 (3)	0.0454 (18)	0.0289 (18)	0.0079 (15)	0.0206 (18)
C7	0.0505 (15)	0.0534 (16)	0.0440 (16)	0.0213 (13)	0.0122 (13)	0.0137 (14)
C8	0.118 (3)	0.107 (3)	0.048 (2)	0.028 (3)	0.012 (2)	0.028 (2)
C9	0.072 (2)	0.071 (2)	0.099 (3)	0.0195 (18)	0.045 (2)	0.040 (2)
C10	0.0311 (11)	0.0372 (12)	0.0356 (13)	0.0080 (10)	0.0124 (10)	0.0086 (11)
C11	0.0314 (11)	0.0427 (13)	0.0322 (13)	0.0096 (10)	0.0092 (10)	0.0074 (11)
C12	0.0408 (13)	0.0528 (15)	0.0388 (14)	0.0160 (12)	0.0138 (12)	0.0162 (12)
C13	0.0509 (16)	0.082 (2)	0.0415 (16)	0.0228 (16)	0.0248 (14)	0.0190 (15)
C14	0.0529 (16)	0.072 (2)	0.0452 (17)	0.0309 (16)	0.0190 (14)	0.0088 (15)
C15	0.0667 (19)	0.0572 (18)	0.061 (2)	0.0364 (16)	0.0254 (17)	0.0173 (16)
C16	0.0573 (16)	0.0519 (16)	0.0507 (17)	0.0278 (14)	0.0251 (14)	0.0200 (14)
C17	0.099 (3)	0.055 (2)	0.162 (4)	0.043 (2)	0.062 (3)	0.033 (2)
C18	0.136 (4)	0.153 (4)	0.069 (3)	0.084 (4)	0.001 (3)	0.024 (3)
C19	0.0367 (12)	0.0344 (12)	0.0424 (14)	0.0154 (10)	0.0147 (11)	0.0146 (11)
C20	0.0382 (12)	0.0345 (12)	0.0535 (16)	0.0162 (10)	0.0146 (12)	0.0205 (12)
C21	0.0438 (14)	0.0557 (17)	0.0637 (19)	0.0206 (13)	0.0220 (14)	0.0303 (15)
C22	0.0405 (15)	0.070 (2)	0.088 (3)	0.0191 (15)	0.0245 (17)	0.041 (2)
C23	0.0399 (16)	0.075 (2)	0.106 (3)	0.0065 (16)	0.0073 (19)	0.043 (2)
C24	0.062 (2)	0.101 (3)	0.064 (2)	0.004 (2)	−0.0074 (19)	0.027 (2)
C25	0.0489 (17)	0.075 (2)	0.059 (2)	0.0078 (16)	0.0113 (15)	0.0283 (18)
C26	0.088 (3)	0.065 (2)	0.121 (3)	0.035 (2)	0.044 (3)	0.054 (2)
C27	0.094 (3)	0.081 (3)	0.055 (2)	0.016 (2)	−0.002 (2)	0.019 (2)
C28	0.0456 (14)	0.0376 (14)	0.079 (2)	0.0178 (12)	0.0311 (15)	0.0211 (14)
C29	0.0422 (15)	0.0523 (17)	0.100 (3)	0.0246 (14)	0.0323 (17)	0.0353 (18)
C30	0.0329 (13)	0.0598 (19)	0.101 (3)	0.0149 (13)	0.0197 (16)	0.0427 (19)
C31	0.0332 (12)	0.0439 (14)	0.0652 (19)	0.0074 (11)	0.0101 (13)	0.0264 (14)
C32	0.0344 (12)	0.0326 (12)	0.0512 (15)	0.0095 (10)	0.0134 (11)	0.0214 (12)
C33	0.0389 (14)	0.0508 (17)	0.072 (2)	0.0028 (13)	0.0012 (15)	0.0174 (16)
C34	0.0537 (17)	0.0441 (16)	0.060 (2)	0.0029 (14)	0.0032 (15)	0.0064 (15)
C35	0.0492 (15)	0.0355 (13)	0.0512 (17)	0.0115 (12)	0.0104 (13)	0.0100 (13)
C36	0.0371 (12)	0.0320 (12)	0.0435 (14)	0.0115 (10)	0.0111 (11)	0.0153 (11)
C37	0.072 (2)	0.0389 (15)	0.059 (2)	0.0192 (15)	0.0191 (17)	0.0045 (14)
C38	0.0657 (19)	0.0486 (16)	0.072 (2)	0.0317 (15)	0.0310 (18)	0.0190 (16)
C39	0.0447 (14)	0.0425 (14)	0.0588 (18)	0.0208 (12)	0.0177 (14)	0.0177 (13)
C40	0.0411 (13)	0.0375 (13)	0.0441 (15)	0.0160 (11)	0.0172 (12)	0.0139 (12)
C41	0.0438 (14)	0.0401 (14)	0.0556 (17)	0.0165 (12)	0.0146 (13)	0.0216 (13)
C42	0.0481 (16)	0.069 (2)	0.076 (2)	0.0231 (15)	0.0239 (16)	0.0356 (18)
C43	0.0495 (18)	0.081 (2)	0.119 (3)	0.0286 (18)	0.032 (2)	0.053 (3)
C44	0.0488 (19)	0.077 (3)	0.132 (4)	0.0105 (18)	0.008 (2)	0.049 (3)
C45	0.069 (2)	0.091 (3)	0.081 (3)	0.002 (2)	−0.010 (2)	0.023 (2)
C46	0.0555 (18)	0.077 (2)	0.061 (2)	0.0091 (17)	0.0099 (17)	0.0234 (18)
C47	0.168 (5)	0.136 (4)	0.173 (5)	0.104 (4)	0.105 (4)	0.119 (4)
C48	0.097 (3)	0.096 (3)	0.104 (3)	0.036 (2)	0.045 (3)	−0.005 (3)
C49	0.0426 (13)	0.0359 (12)	0.0347 (13)	0.0173 (11)	0.0152 (11)	0.0133 (11)
C50	0.0445 (13)	0.0403 (13)	0.0400 (14)	0.0200 (11)	0.0167 (12)	0.0176 (12)
C51	0.073 (2)	0.0530 (18)	0.071 (2)	0.0341 (16)	0.0138 (18)	0.0201 (17)
C52	0.088 (3)	0.078 (2)	0.099 (3)	0.058 (2)	0.027 (2)	0.046 (2)
C53	0.069 (2)	0.109 (3)	0.075 (3)	0.050 (2)	0.018 (2)	0.054 (2)
C54	0.060 (2)	0.091 (3)	0.060 (2)	0.028 (2)	0.0001 (17)	0.023 (2)
C55	0.0550 (17)	0.0568 (17)	0.0498 (18)	0.0231 (14)	0.0058 (14)	0.0151 (15)
C56	0.130 (4)	0.109 (3)	0.047 (2)	0.040 (3)	0.007 (2)	0.023 (2)
C57	0.091 (3)	0.093 (3)	0.112 (3)	0.028 (2)	0.051 (3)	0.053 (3)
C58	0.0375 (12)	0.0464 (14)	0.0348 (14)	0.0114 (11)	0.0156 (11)	0.0106 (12)
C59	0.0354 (12)	0.0418 (14)	0.0354 (13)	0.0101 (11)	0.0122 (11)	0.0075 (11)
C60	0.0459 (15)	0.0639 (18)	0.0391 (15)	0.0193 (13)	0.0163 (13)	0.0166 (13)
C61	0.0563 (17)	0.089 (2)	0.0421 (17)	0.0271 (17)	0.0241 (15)	0.0210 (17)
C62	0.0629 (19)	0.087 (2)	0.0496 (18)	0.0402 (18)	0.0258 (16)	0.0125 (17)
C63	0.077 (2)	0.0623 (19)	0.069 (2)	0.0397 (17)	0.0334 (19)	0.0219 (17)
C64	0.0655 (18)	0.0598 (18)	0.0565 (19)	0.0293 (15)	0.0332 (16)	0.0239 (15)
C65	0.190 (5)	0.127 (4)	0.137 (4)	0.089 (4)	0.110 (4)	0.101 (3)
C66	0.056 (2)	0.136 (4)	0.104 (3)	0.029 (2)	0.007 (2)	−0.004 (3)
C67	0.0474 (15)	0.0443 (15)	0.083 (2)	0.0172 (13)	0.0280 (16)	0.0228 (15)
C68	0.0518 (18)	0.061 (2)	0.109 (3)	0.0261 (16)	0.038 (2)	0.040 (2)
C69	0.0366 (15)	0.067 (2)	0.108 (3)	0.0106 (15)	0.0185 (18)	0.039 (2)
C70	0.0437 (15)	0.0491 (16)	0.069 (2)	0.0064 (13)	0.0127 (15)	0.0239 (16)
C71	0.0399 (13)	0.0362 (13)	0.0541 (17)	0.0082 (11)	0.0116 (13)	0.0195 (13)
C72	0.0473 (17)	0.058 (2)	0.087 (3)	−0.0051 (15)	−0.0003 (18)	0.0111 (19)
C73	0.063 (2)	0.0474 (18)	0.079 (3)	−0.0002 (16)	0.0062 (19)	−0.0003 (17)
C74	0.0578 (17)	0.0393 (15)	0.0523 (18)	0.0103 (13)	0.0086 (15)	0.0090 (14)
C75	0.0450 (13)	0.0321 (12)	0.0425 (15)	0.0094 (11)	0.0103 (12)	0.0147 (11)
C76	0.081 (2)	0.0403 (16)	0.064 (2)	0.0211 (16)	0.0129 (19)	0.0017 (15)
C77	0.078 (2)	0.0513 (18)	0.066 (2)	0.0363 (17)	0.0161 (19)	0.0067 (16)
C78	0.0558 (17)	0.0481 (16)	0.062 (2)	0.0275 (14)	0.0067 (15)	0.0092 (15)

### Geometric parameters (Å, º)

**Table:**

Nd1—O1	2.3986 (16)	C20—C25	1.380 (4)
Nd1—O2	2.3879 (16)	C21—H21	0.9300
Nd1—O5	2.4202 (17)	C21—C22	1.380 (4)
Nd1—O6	2.3952 (17)	C22—H22	0.9300
Nd1—O9	2.4197 (17)	C22—C23	1.356 (5)
Nd1—O10	2.4065 (15)	C23—H23	0.9300
Nd1—N4	2.6725 (19)	C23—C24	1.374 (5)
Nd1—N5	2.6579 (19)	C24—H24	0.9300
Nd2—O13	2.4291 (17)	C24—C25	1.390 (4)
Nd2—O14	2.4014 (15)	C25—H25	0.9300
Nd2—O17	2.4063 (17)	C26—H26A	0.9600
Nd2—O18	2.3705 (17)	C26—H26B	0.9600
Nd2—O21	2.4282 (17)	C26—H26C	0.9600
Nd2—O22	2.3931 (16)	C27—H27A	0.9600
Nd2—N9	2.665 (2)	C27—H27B	0.9600
Nd2—N10	2.669 (2)	C27—H27C	0.9600
P1—O1	1.4907 (17)	C28—H28	0.9300
P1—O3	1.571 (2)	C28—C29	1.384 (4)
P1—O4	1.5754 (18)	C29—H29	0.9300
P1—N1	1.610 (2)	C29—C30	1.355 (4)
P2—O5	1.4814 (17)	C30—H30	0.9300
P2—O7	1.5771 (19)	C30—C31	1.405 (4)
P2—O8	1.565 (2)	C31—C32	1.413 (3)
P2—N2	1.604 (2)	C31—C33	1.435 (4)
P3—O9	1.4847 (16)	C32—C36	1.438 (3)
P3—O11	1.5786 (19)	C33—H33	0.9300
P3—O12	1.567 (2)	C33—C34	1.324 (4)
P3—N3	1.609 (2)	C34—H34	0.9300
P4—O13	1.4835 (17)	C34—C35	1.433 (4)
P4—O15	1.572 (2)	C35—C36	1.406 (3)
P4—O16	1.566 (2)	C35—C37	1.405 (4)
P4—N6	1.613 (2)	C37—H37	0.9300
P5—O17	1.4897 (18)	C37—C38	1.356 (4)
P5—O19	1.570 (2)	C38—H38	0.9300
P5—O20	1.5730 (19)	C38—C39	1.392 (4)
P5—N7	1.604 (2)	C39—H39	0.9300
P6—O21	1.4804 (17)	C40—C41	1.493 (3)
P6—O23	1.574 (2)	C41—C42	1.384 (3)
P6—O24	1.568 (2)	C41—C46	1.373 (4)
P6—N8	1.604 (2)	C42—H42	0.9300
O2—C1	1.252 (3)	C42—C43	1.383 (4)
O3—C8	1.425 (4)	C43—H43	0.9300
O4—C9	1.428 (3)	C43—C44	1.351 (5)
O6—C10	1.258 (3)	C44—H44	0.9300
O7—C17	1.460 (3)	C44—C45	1.381 (5)
O8—C18	1.399 (4)	C45—H45	0.9300
O10—C19	1.267 (2)	C45—C46	1.392 (5)
O11—C26	1.441 (4)	C46—H46	0.9300
O12—C27	1.426 (4)	C47—H47A	0.9600
O14—C40	1.268 (3)	C47—H47B	0.9600
O15—C47	1.403 (4)	C47—H47C	0.9600
O16—C48	1.424 (4)	C48—H48A	0.9600
O18—C49	1.254 (3)	C48—H48B	0.9600
O19—C56	1.420 (4)	C48—H48C	0.9600
O20—C57	1.436 (4)	C49—C50	1.490 (3)
O22—C58	1.253 (3)	C50—C51	1.382 (4)
O23—C65	1.433 (4)	C50—C55	1.376 (4)
O24—C66	1.452 (4)	C51—H51	0.9300
N1—C1	1.316 (3)	C51—C52	1.393 (5)
N2—C10	1.316 (3)	C52—H52	0.9300
N3—C19	1.316 (3)	C52—C53	1.344 (5)
N4—C36	1.358 (3)	C53—H53	0.9300
N4—C39	1.321 (3)	C53—C54	1.362 (5)
N5—C28	1.331 (3)	C54—H54	0.9300
N5—C32	1.360 (3)	C54—C55	1.382 (4)
N6—C40	1.315 (3)	C55—H55	0.9300
N7—C49	1.315 (3)	C56—H56A	0.9600
N8—C58	1.308 (3)	C56—H56B	0.9600
N9—C67	1.326 (3)	C56—H56C	0.9600
N9—C71	1.355 (3)	C57—H57A	0.9600
N10—C75	1.359 (3)	C57—H57B	0.9600
N10—C78	1.319 (3)	C57—H57C	0.9600
C1—C2	1.493 (3)	C58—C59	1.508 (3)
C2—C3	1.385 (3)	C59—C60	1.383 (3)
C2—C7	1.384 (4)	C59—C64	1.370 (4)
C3—H3	0.9300	C60—H60	0.9300
C3—C4	1.391 (4)	C60—C61	1.393 (4)
C4—H4	0.9300	C61—H61	0.9300
C4—C5	1.354 (5)	C61—C62	1.362 (4)
C5—H5	0.9300	C62—H62	0.9300
C5—C6	1.377 (5)	C62—C63	1.366 (4)
C6—H6	0.9300	C63—H63	0.9300
C6—C7	1.385 (4)	C63—C64	1.394 (4)
C7—H7	0.9300	C64—H64	0.9300
C8—H8A	0.9600	C65—H65A	0.9600
C8—H8B	0.9600	C65—H65B	0.9600
C8—H8C	0.9600	C65—H65C	0.9600
C9—H9A	0.9600	C66—H66A	0.9600
C9—H9B	0.9600	C66—H66B	0.9600
C9—H9C	0.9600	C66—H66C	0.9600
C10—C11	1.510 (3)	C67—H67	0.9300
C11—C12	1.384 (3)	C67—C68	1.394 (4)
C11—C16	1.380 (3)	C68—H68	0.9300
C12—H12	0.9300	C68—C69	1.346 (5)
C12—C13	1.396 (3)	C69—H69	0.9300
C13—H13	0.9300	C69—C70	1.397 (4)
C13—C14	1.374 (4)	C70—C71	1.408 (4)
C14—H14	0.9300	C70—C72	1.445 (4)
C14—C15	1.360 (4)	C71—C75	1.438 (3)
C15—H15	0.9300	C72—H72	0.9300
C15—C16	1.390 (3)	C72—C73	1.337 (5)
C16—H16	0.9300	C73—H73	0.9300
C17—H17A	0.9600	C73—C74	1.425 (4)
C17—H17B	0.9600	C74—C75	1.406 (3)
C17—H17C	0.9600	C74—C76	1.402 (4)
C18—H18A	0.9600	C76—H76	0.9300
C18—H18B	0.9600	C76—C77	1.349 (4)
C18—H18C	0.9600	C77—H77	0.9300
C19—C20	1.496 (3)	C77—C78	1.398 (4)
C20—C21	1.386 (3)	C78—H78	0.9300
			
O1—Nd1—O5	105.89 (7)	N3—C19—C20	116.0 (2)
O1—Nd1—O9	95.87 (6)	C21—C20—C19	121.7 (2)
O1—Nd1—O10	74.75 (6)	C25—C20—C19	120.1 (2)
O1—Nd1—N4	154.43 (6)	C25—C20—C21	118.3 (3)
O1—Nd1—N5	141.74 (6)	C20—C21—H21	119.4
O2—Nd1—O1	72.41 (6)	C22—C21—C20	121.3 (3)
O2—Nd1—O5	145.25 (6)	C22—C21—H21	119.4
O2—Nd1—O6	74.64 (7)	C21—C22—H22	120.1
O2—Nd1—O9	74.16 (6)	C23—C22—C21	119.8 (3)
O2—Nd1—O10	130.12 (6)	C23—C22—H22	120.1
O2—Nd1—N4	122.25 (6)	C22—C23—H23	119.8
O2—Nd1—N5	71.56 (6)	C22—C23—C24	120.4 (3)
O5—Nd1—N4	74.38 (6)	C24—C23—H23	119.8
O5—Nd1—N5	96.51 (7)	C23—C24—H24	120.0
O6—Nd1—O1	80.63 (7)	C23—C24—C25	120.1 (3)
O6—Nd1—O5	70.92 (6)	C25—C24—H24	120.0
O6—Nd1—O9	148.15 (6)	C20—C25—C24	120.2 (3)
O6—Nd1—O10	134.46 (7)	C20—C25—H25	119.9
O6—Nd1—N4	121.96 (6)	C24—C25—H25	119.9
O6—Nd1—N5	77.92 (7)	O11—C26—H26A	109.5
O9—Nd1—O5	139.08 (6)	O11—C26—H26B	109.5
O9—Nd1—N4	71.37 (6)	O11—C26—H26C	109.5
O9—Nd1—N5	86.35 (6)	H26A—C26—H26B	109.5
O10—Nd1—O5	79.75 (6)	H26A—C26—H26C	109.5
O10—Nd1—O9	73.04 (6)	H26B—C26—H26C	109.5
O10—Nd1—N4	80.25 (6)	O12—C27—H27A	109.5
O10—Nd1—N5	140.82 (6)	O12—C27—H27B	109.5
N5—Nd1—N4	61.46 (6)	O12—C27—H27C	109.5
O13—Nd2—N9	84.61 (6)	H27A—C27—H27B	109.5
O13—Nd2—N10	71.48 (6)	H27A—C27—H27C	109.5
O14—Nd2—O13	73.51 (6)	H27B—C27—H27C	109.5
O14—Nd2—O17	74.69 (6)	N5—C28—H28	118.1
O14—Nd2—O21	79.65 (6)	N5—C28—C29	123.8 (3)
O14—Nd2—N9	140.86 (6)	C29—C28—H28	118.1
O14—Nd2—N10	80.91 (6)	C28—C29—H29	120.5
O17—Nd2—O13	100.04 (6)	C30—C29—C28	119.1 (3)
O17—Nd2—O21	102.53 (7)	C30—C29—H29	120.5
O17—Nd2—N9	142.29 (6)	C29—C30—H30	119.9
O17—Nd2—N10	155.58 (6)	C29—C30—C31	120.2 (3)
O18—Nd2—O13	74.25 (6)	C31—C30—H30	119.9
O18—Nd2—O14	128.24 (6)	C30—C31—C32	117.0 (3)
O18—Nd2—O17	72.40 (6)	C30—C31—C33	123.9 (3)
O18—Nd2—O21	145.98 (6)	C32—C31—C33	119.1 (2)
O18—Nd2—O22	74.53 (7)	N5—C32—C31	122.5 (2)
O18—Nd2—N9	73.04 (6)	N5—C32—C36	118.4 (2)
O18—Nd2—N10	124.25 (6)	C31—C32—C36	119.2 (2)
O21—Nd2—O13	138.63 (6)	C31—C33—H33	119.2
O21—Nd2—N9	97.66 (6)	C34—C33—C31	121.5 (3)
O21—Nd2—N10	73.68 (6)	C34—C33—H33	119.2
O22—Nd2—O13	147.55 (7)	C33—C34—H34	119.3
O22—Nd2—O14	134.97 (7)	C33—C34—C35	121.3 (3)
O22—Nd2—O17	78.67 (7)	C35—C34—H34	119.3
O22—Nd2—O21	71.51 (6)	C36—C35—C34	119.4 (2)
O22—Nd2—N9	78.03 (8)	C37—C35—C34	123.4 (3)
O22—Nd2—N10	120.99 (7)	C37—C35—C36	117.1 (3)
N9—Nd2—N10	61.26 (6)	N4—C36—C32	118.1 (2)
O1—P1—O3	112.66 (12)	N4—C36—C35	122.4 (2)
O1—P1—O4	109.62 (10)	C35—C36—C32	119.5 (2)
O1—P1—N1	118.72 (11)	C35—C37—H37	119.9
O3—P1—O4	100.68 (11)	C38—C37—C35	120.1 (3)
O3—P1—N1	106.13 (12)	C38—C37—H37	119.9
O4—P1—N1	107.41 (11)	C37—C38—H38	120.7
O5—P2—O7	111.66 (12)	C37—C38—C39	118.6 (3)
O5—P2—O8	106.29 (11)	C39—C38—H38	120.7
O5—P2—N2	120.50 (11)	N4—C39—C38	123.8 (3)
O7—P2—N2	102.81 (11)	N4—C39—H39	118.1
O8—P2—O7	104.54 (12)	C38—C39—H39	118.1
O8—P2—N2	110.00 (12)	O14—C40—N6	126.2 (2)
O9—P3—O11	110.11 (10)	O14—C40—C41	117.4 (2)
O9—P3—O12	113.09 (11)	N6—C40—C41	116.3 (2)
O9—P3—N3	118.33 (11)	C42—C41—C40	121.3 (3)
O11—P3—N3	107.04 (11)	C46—C41—C40	119.8 (2)
O12—P3—O11	101.09 (12)	C46—C41—C42	118.9 (3)
O12—P3—N3	105.63 (12)	C41—C42—H42	119.7
O13—P4—O15	111.05 (11)	C43—C42—C41	120.7 (3)
O13—P4—O16	108.68 (12)	C43—C42—H42	119.7
O13—P4—N6	118.23 (11)	C42—C43—H43	120.0
O15—P4—N6	106.37 (12)	C44—C43—C42	120.0 (3)
O16—P4—O15	101.28 (13)	C44—C43—H43	120.0
O16—P4—N6	109.91 (12)	C43—C44—H44	119.7
O17—P5—O19	112.78 (12)	C43—C44—C45	120.6 (4)
O17—P5—O20	110.18 (11)	C45—C44—H44	119.7
O17—P5—N7	119.02 (11)	C44—C45—H45	120.3
O19—P5—O20	100.54 (12)	C44—C45—C46	119.3 (4)
O19—P5—N7	105.20 (13)	C46—C45—H45	120.3
O20—P5—N7	107.40 (12)	C41—C46—C45	120.5 (3)
O21—P6—O23	108.90 (12)	C41—C46—H46	119.8
O21—P6—O24	111.59 (12)	C45—C46—H46	119.8
O21—P6—N8	119.17 (11)	O15—C47—H47A	109.5
O23—P6—N8	107.56 (12)	O15—C47—H47B	109.5
O24—P6—O23	100.34 (13)	O15—C47—H47C	109.5
O24—P6—N8	107.58 (12)	H47A—C47—H47B	109.5
P1—O1—Nd1	130.79 (10)	H47A—C47—H47C	109.5
C1—O2—Nd1	141.14 (15)	H47B—C47—H47C	109.5
C8—O3—P1	120.0 (2)	O16—C48—H48A	109.5
C9—O4—P1	119.33 (19)	O16—C48—H48B	109.5
P2—O5—Nd1	134.60 (10)	O16—C48—H48C	109.5
C10—O6—Nd1	144.24 (16)	H48A—C48—H48B	109.5
C17—O7—P2	119.52 (18)	H48A—C48—H48C	109.5
C18—O8—P2	122.5 (2)	H48B—C48—H48C	109.5
P3—O9—Nd1	131.27 (10)	O18—C49—N7	126.7 (2)
C19—O10—Nd1	131.91 (14)	O18—C49—C50	115.5 (2)
C26—O11—P3	118.2 (2)	N7—C49—C50	117.8 (2)
C27—O12—P3	121.0 (2)	C51—C50—C49	122.8 (3)
P4—O13—Nd2	129.42 (11)	C55—C50—C49	119.2 (2)
C40—O14—Nd2	132.16 (15)	C55—C50—C51	118.0 (3)
C47—O15—P4	119.7 (2)	C50—C51—H51	120.0
C48—O16—P4	122.4 (2)	C50—C51—C52	120.0 (3)
P5—O17—Nd2	131.76 (10)	C52—C51—H51	120.0
C49—O18—Nd2	142.25 (16)	C51—C52—H52	119.6
C56—O19—P5	120.6 (2)	C53—C52—C51	120.9 (3)
C57—O20—P5	119.3 (2)	C53—C52—H52	119.6
P6—O21—Nd2	134.70 (10)	C52—C53—H53	120.0
C58—O22—Nd2	143.54 (17)	C52—C53—C54	120.0 (3)
C65—O23—P6	120.8 (2)	C54—C53—H53	120.0
C66—O24—P6	117.5 (2)	C53—C54—H54	120.0
C1—N1—P1	120.83 (17)	C53—C54—C55	120.0 (3)
C10—N2—P2	122.01 (17)	C55—C54—H54	120.0
C19—N3—P3	121.89 (17)	C50—C55—C54	121.1 (3)
C36—N4—Nd1	119.87 (14)	C50—C55—H55	119.4
C39—N4—Nd1	121.42 (16)	C54—C55—H55	119.4
C39—N4—C36	117.8 (2)	O19—C56—H56A	109.5
C28—N5—Nd1	121.43 (17)	O19—C56—H56B	109.5
C28—N5—C32	117.5 (2)	O19—C56—H56C	109.5
C32—N5—Nd1	120.09 (14)	H56A—C56—H56B	109.5
C40—N6—P4	121.49 (18)	H56A—C56—H56C	109.5
C49—N7—P5	122.03 (18)	H56B—C56—H56C	109.5
C58—N8—P6	123.71 (18)	O20—C57—H57A	109.5
C67—N9—Nd2	121.28 (18)	O20—C57—H57B	109.5
C67—N9—C71	118.0 (2)	O20—C57—H57C	109.5
C71—N9—Nd2	120.03 (15)	H57A—C57—H57B	109.5
C75—N10—Nd2	119.96 (15)	H57A—C57—H57C	109.5
C78—N10—Nd2	121.64 (18)	H57B—C57—H57C	109.5
C78—N10—C75	117.5 (2)	O22—C58—N8	127.1 (2)
O2—C1—N1	126.8 (2)	O22—C58—C59	115.2 (2)
O2—C1—C2	115.3 (2)	N8—C58—C59	117.7 (2)
N1—C1—C2	117.9 (2)	C60—C59—C58	120.9 (2)
C3—C2—C1	121.9 (2)	C64—C59—C58	119.6 (2)
C7—C2—C1	119.8 (2)	C64—C59—C60	119.5 (2)
C7—C2—C3	118.3 (3)	C59—C60—H60	120.3
C2—C3—H3	119.9	C59—C60—C61	119.5 (3)
C2—C3—C4	120.3 (3)	C61—C60—H60	120.3
C4—C3—H3	119.9	C60—C61—H61	119.9
C3—C4—H4	119.7	C62—C61—C60	120.3 (3)
C5—C4—C3	120.7 (3)	C62—C61—H61	119.9
C5—C4—H4	119.7	C61—C62—H62	119.7
C4—C5—H5	120.0	C61—C62—C63	120.7 (3)
C4—C5—C6	120.0 (3)	C63—C62—H62	119.7
C6—C5—H5	120.0	C62—C63—H63	120.3
C5—C6—H6	120.1	C62—C63—C64	119.4 (3)
C5—C6—C7	119.8 (3)	C64—C63—H63	120.3
C7—C6—H6	120.1	C59—C64—C63	120.6 (3)
C2—C7—C6	120.9 (3)	C59—C64—H64	119.7
C2—C7—H7	119.6	C63—C64—H64	119.7
C6—C7—H7	119.6	O23—C65—H65A	109.5
O3—C8—H8A	109.5	O23—C65—H65B	109.5
O3—C8—H8B	109.5	O23—C65—H65C	109.5
O3—C8—H8C	109.5	H65A—C65—H65B	109.5
H8A—C8—H8B	109.5	H65A—C65—H65C	109.5
H8A—C8—H8C	109.5	H65B—C65—H65C	109.5
H8B—C8—H8C	109.5	O24—C66—H66A	109.5
O4—C9—H9A	109.5	O24—C66—H66B	109.5
O4—C9—H9B	109.5	O24—C66—H66C	109.5
O4—C9—H9C	109.5	H66A—C66—H66B	109.5
H9A—C9—H9B	109.5	H66A—C66—H66C	109.5
H9A—C9—H9C	109.5	H66B—C66—H66C	109.5
H9B—C9—H9C	109.5	N9—C67—H67	118.4
O6—C10—N2	126.9 (2)	N9—C67—C68	123.3 (3)
O6—C10—C11	115.1 (2)	C68—C67—H67	118.4
N2—C10—C11	117.9 (2)	C67—C68—H68	120.5
C12—C11—C10	121.2 (2)	C69—C68—C67	119.0 (3)
C16—C11—C10	119.0 (2)	C69—C68—H68	120.5
C16—C11—C12	119.8 (2)	C68—C69—H69	119.9
C11—C12—H12	120.2	C68—C69—C70	120.2 (3)
C11—C12—C13	119.6 (3)	C70—C69—H69	119.9
C13—C12—H12	120.2	C69—C70—C71	117.5 (3)
C12—C13—H13	119.9	C69—C70—C72	123.9 (3)
C14—C13—C12	120.1 (2)	C71—C70—C72	118.5 (3)
C14—C13—H13	119.9	N9—C71—C70	122.0 (2)
C13—C14—H14	120.0	N9—C71—C75	118.3 (2)
C15—C14—C13	120.0 (2)	C70—C71—C75	119.6 (2)
C15—C14—H14	120.0	C70—C72—H72	119.2
C14—C15—H15	119.6	C73—C72—C70	121.6 (3)
C14—C15—C16	120.8 (3)	C73—C72—H72	119.2
C16—C15—H15	119.6	C72—C73—H73	119.5
C11—C16—C15	119.6 (2)	C72—C73—C74	121.0 (3)
C11—C16—H16	120.2	C74—C73—H73	119.5
C15—C16—H16	120.2	C75—C74—C73	119.7 (3)
O7—C17—H17A	109.5	C76—C74—C73	123.1 (3)
O7—C17—H17B	109.5	C76—C74—C75	117.2 (3)
O7—C17—H17C	109.5	N10—C75—C71	117.9 (2)
H17A—C17—H17B	109.5	N10—C75—C74	122.5 (2)
H17A—C17—H17C	109.5	C74—C75—C71	119.5 (2)
H17B—C17—H17C	109.5	C74—C76—H76	119.9
O8—C18—H18A	109.5	C77—C76—C74	120.2 (3)
O8—C18—H18B	109.5	C77—C76—H76	119.9
O8—C18—H18C	109.5	C76—C77—H77	120.7
H18A—C18—H18B	109.5	C76—C77—C78	118.7 (3)
H18A—C18—H18C	109.5	C78—C77—H77	120.7
H18B—C18—H18C	109.5	N10—C78—C77	123.8 (3)
O10—C19—N3	126.9 (2)	N10—C78—H78	118.1
O10—C19—C20	117.1 (2)	C77—C78—H78	118.1
			
Nd1—O2—C1—N1	25.5 (4)	O21—P6—O24—C66	−62.5 (3)
Nd1—O2—C1—C2	−154.29 (19)	O21—P6—N8—C58	−1.7 (3)
Nd1—O6—C10—N2	−8.2 (5)	O22—Nd2—O13—P4	−152.75 (14)
Nd1—O6—C10—C11	170.5 (2)	O22—Nd2—O14—C40	121.1 (2)
Nd1—O10—C19—N3	48.2 (3)	O22—Nd2—O17—P5	65.20 (15)
Nd1—O10—C19—C20	−131.51 (18)	O22—Nd2—O18—C49	−96.4 (3)
Nd1—N4—C36—C32	11.1 (3)	O22—Nd2—O21—P6	4.99 (16)
Nd1—N4—C36—C35	−168.95 (19)	O22—Nd2—N9—C67	−41.0 (2)
Nd1—N4—C39—C38	169.5 (2)	O22—Nd2—N9—C71	148.9 (2)
Nd1—N5—C28—C29	−169.0 (2)	O22—Nd2—N10—C75	−65.7 (2)
Nd1—N5—C32—C31	168.07 (18)	O22—Nd2—N10—C78	125.2 (2)
Nd1—N5—C32—C36	−12.3 (3)	O22—C58—C59—C60	−160.5 (3)
Nd2—O14—C40—N6	46.4 (3)	O22—C58—C59—C64	17.4 (4)
Nd2—O14—C40—C41	−133.99 (19)	O23—P6—O21—Nd2	−127.79 (15)
Nd2—O18—C49—N7	21.5 (4)	O23—P6—O24—C66	−177.7 (2)
Nd2—O18—C49—C50	−158.51 (19)	O23—P6—N8—C58	122.7 (2)
Nd2—O22—C58—N8	0.4 (5)	O24—P6—O21—Nd2	122.37 (16)
Nd2—O22—C58—C59	179.9 (2)	O24—P6—O23—C65	−64.9 (3)
Nd2—N9—C67—C68	−170.4 (2)	O24—P6—N8—C58	−130.0 (2)
Nd2—N9—C71—C70	168.9 (2)	N1—P1—O1—Nd1	34.00 (18)
Nd2—N9—C71—C75	−12.9 (3)	N1—P1—O3—C8	−173.4 (3)
Nd2—N10—C75—C71	12.5 (3)	N1—P1—O4—C9	79.0 (2)
Nd2—N10—C75—C74	−167.80 (19)	N1—C1—C2—C3	21.5 (3)
Nd2—N10—C78—C77	169.3 (2)	N1—C1—C2—C7	−158.7 (2)
P1—N1—C1—O2	0.1 (4)	N2—P2—O5—Nd1	3.5 (2)
P1—N1—C1—C2	179.85 (16)	N2—P2—O7—C17	−179.5 (3)
P2—N2—C10—O6	−2.2 (4)	N2—P2—O8—C18	−56.7 (3)
P2—N2—C10—C11	179.21 (17)	N2—C10—C11—C12	11.8 (3)
P3—N3—C19—O10	−5.7 (4)	N2—C10—C11—C16	−170.1 (2)
P3—N3—C19—C20	173.98 (17)	N3—P3—O9—Nd1	19.29 (19)
P4—N6—C40—O14	−0.4 (4)	N3—P3—O11—C26	75.2 (2)
P4—N6—C40—C41	−179.96 (17)	N3—P3—O12—C27	179.0 (2)
P5—N7—C49—O18	−1.2 (4)	N3—C19—C20—C21	18.6 (3)
P5—N7—C49—C50	178.82 (17)	N3—C19—C20—C25	−161.0 (3)
P6—N8—C58—O22	3.7 (4)	N4—Nd1—O1—P1	−143.87 (13)
P6—N8—C58—C59	−175.78 (18)	N4—Nd1—O2—C1	140.1 (3)
O1—Nd1—O2—C1	−16.6 (3)	N4—Nd1—O5—P2	125.84 (18)
O1—Nd1—O5—P2	−80.75 (18)	N4—Nd1—O6—C10	−45.9 (3)
O1—Nd1—O6—C10	121.1 (3)	N4—Nd1—O9—P3	90.03 (15)
O1—Nd1—O9—P3	−67.22 (14)	N4—Nd1—O10—C19	−113.3 (2)
O1—Nd1—O10—C19	61.2 (2)	N4—Nd1—N5—C28	−179.3 (2)
O1—Nd1—N4—C36	147.36 (16)	N4—Nd1—N5—C32	12.15 (16)
O1—Nd1—N4—C39	−21.6 (3)	N5—Nd1—O1—P1	5.7 (2)
O1—Nd1—N5—C28	15.0 (3)	N5—Nd1—O2—C1	176.4 (3)
O1—Nd1—N5—C32	−153.46 (16)	N5—Nd1—O5—P2	67.92 (18)
O1—P1—O3—C8	−41.9 (3)	N5—Nd1—O6—C10	−90.8 (3)
O1—P1—O4—C9	−51.3 (2)	N5—Nd1—O9—P3	151.11 (14)
O1—P1—N1—C1	−27.1 (2)	N5—Nd1—O10—C19	−101.3 (2)
O2—Nd1—O1—P1	−14.50 (13)	N5—Nd1—N4—C36	−11.76 (16)
O2—Nd1—O5—P2	1.1 (3)	N5—Nd1—N4—C39	179.3 (2)
O2—Nd1—O6—C10	−164.7 (3)	N5—C28—C29—C30	1.0 (5)
O2—Nd1—O9—P3	−137.04 (15)	N5—C32—C36—N4	0.7 (3)
O2—Nd1—O10—C19	11.0 (2)	N5—C32—C36—C35	−179.3 (2)
O2—Nd1—N4—C36	27.97 (19)	N6—P4—O13—Nd2	25.1 (2)
O2—Nd1—N4—C39	−141.00 (18)	N6—P4—O15—C47	78.6 (3)
O2—Nd1—N5—C28	35.4 (2)	N6—P4—O16—C48	26.4 (3)
O2—Nd1—N5—C32	−133.11 (19)	N6—C40—C41—C42	24.0 (4)
O2—C1—C2—C3	−158.7 (2)	N6—C40—C41—C46	−155.4 (3)
O2—C1—C2—C7	21.1 (3)	N7—P5—O17—Nd2	27.0 (2)
O3—P1—O1—Nd1	−90.89 (16)	N7—P5—O19—C56	−174.2 (3)
O3—P1—O4—C9	−170.2 (2)	N7—P5—O20—C57	76.7 (3)
O3—P1—N1—C1	100.9 (2)	N7—C49—C50—C51	18.0 (4)
O4—P1—O1—Nd1	157.89 (12)	N7—C49—C50—C55	−160.6 (2)
O4—P1—O3—C8	74.8 (3)	N8—P6—O21—Nd2	−4.0 (2)
O4—P1—N1—C1	−152.10 (19)	N8—P6—O23—C65	47.4 (3)
O5—Nd1—O1—P1	129.20 (13)	N8—P6—O24—C66	70.0 (2)
O5—Nd1—O2—C1	−109.3 (3)	N8—C58—C59—C60	19.1 (4)
O5—Nd1—O6—C10	10.6 (3)	N8—C58—C59—C64	−163.1 (3)
O5—Nd1—O9—P3	55.38 (18)	N9—Nd2—O13—P4	149.69 (15)
O5—Nd1—O10—C19	170.9 (2)	N9—Nd2—O14—C40	−99.1 (2)
O5—Nd1—N4—C36	−118.38 (17)	N9—Nd2—O17—P5	12.4 (2)
O5—Nd1—N4—C39	72.65 (19)	N9—Nd2—O18—C49	−178.3 (3)
O5—Nd1—N5—C28	−111.1 (2)	N9—Nd2—O21—P6	79.59 (17)
O5—Nd1—N5—C32	80.41 (18)	N9—Nd2—O22—C58	−106.2 (3)
O5—P2—O7—C17	−48.9 (3)	N9—Nd2—N10—C75	−12.93 (17)
O5—P2—O8—C18	171.3 (3)	N9—Nd2—N10—C78	178.0 (2)
O5—P2—N2—C10	3.6 (3)	N9—C67—C68—C69	1.4 (5)
O6—Nd1—O1—P1	62.24 (14)	N9—C71—C75—N10	0.3 (4)
O6—Nd1—O2—C1	−101.5 (3)	N9—C71—C75—C74	−179.5 (2)
O6—Nd1—O5—P2	−6.86 (16)	N10—Nd2—O13—P4	88.23 (15)
O6—Nd1—O9—P3	−148.91 (13)	N10—Nd2—O14—C40	−113.6 (2)
O6—Nd1—O10—C19	120.9 (2)	N10—Nd2—O17—P5	−148.81 (14)
O6—Nd1—N4—C36	−63.43 (19)	N10—Nd2—O18—C49	146.4 (3)
O6—Nd1—N4—C39	127.60 (19)	N10—Nd2—O21—P6	136.57 (17)
O6—Nd1—N5—C28	−42.2 (2)	N10—Nd2—O22—C58	−60.6 (4)
O6—Nd1—N5—C32	149.26 (18)	N10—Nd2—N9—C67	−176.8 (2)
O6—C10—C11—C12	−166.9 (2)	N10—Nd2—N9—C71	13.10 (17)
O6—C10—C11—C16	11.2 (3)	C1—C2—C3—C4	178.7 (2)
O7—P2—O5—Nd1	−117.25 (16)	C1—C2—C7—C6	−179.5 (2)
O7—P2—O8—C18	53.1 (3)	C2—C3—C4—C5	1.1 (5)
O7—P2—N2—C10	128.5 (2)	C3—C2—C7—C6	0.3 (4)
O8—P2—O5—Nd1	129.33 (17)	C3—C4—C5—C6	0.0 (5)
O8—P2—O7—C17	65.6 (3)	C4—C5—C6—C7	−0.8 (5)
O8—P2—N2—C10	−120.6 (2)	C5—C6—C7—C2	0.6 (4)
O9—Nd1—O1—P1	−85.80 (14)	C7—C2—C3—C4	−1.2 (4)
O9—Nd1—O2—C1	85.0 (3)	C10—C11—C12—C13	177.0 (2)
O9—Nd1—O5—P2	159.86 (13)	C10—C11—C16—C15	−178.5 (2)
O9—Nd1—O6—C10	−152.8 (3)	C11—C12—C13—C14	1.1 (4)
O9—Nd1—O10—C19	−40.0 (2)	C12—C11—C16—C15	−0.3 (4)
O9—Nd1—N4—C36	84.37 (17)	C12—C13—C14—C15	0.4 (5)
O9—Nd1—N4—C39	−84.60 (19)	C13—C14—C15—C16	−1.9 (5)
O9—Nd1—N5—C28	109.9 (2)	C14—C15—C16—C11	1.8 (5)
O9—Nd1—N5—C32	−58.60 (18)	C16—C11—C12—C13	−1.1 (4)
O9—P3—O11—C26	−54.7 (2)	C19—C20—C21—C22	−179.5 (3)
O9—P3—O12—C27	−50.1 (3)	C19—C20—C25—C24	179.0 (3)
O9—P3—N3—C19	−26.4 (3)	C20—C21—C22—C23	0.1 (5)
O10—Nd1—O1—P1	−156.42 (15)	C21—C20—C25—C24	−0.6 (5)
O10—Nd1—O2—C1	34.5 (3)	C21—C22—C23—C24	0.2 (5)
O10—Nd1—O5—P2	−151.52 (18)	C22—C23—C24—C25	−0.7 (6)
O10—Nd1—O6—C10	63.5 (3)	C23—C24—C25—C20	0.9 (6)
O10—Nd1—O9—P3	4.85 (13)	C25—C20—C21—C22	0.1 (4)
O10—Nd1—N4—C36	159.64 (17)	C28—N5—C32—C31	−0.9 (4)
O10—Nd1—N4—C39	−9.34 (19)	C28—N5—C32—C36	178.8 (2)
O10—Nd1—N5—C28	167.16 (18)	C28—C29—C30—C31	−0.6 (5)
O10—Nd1—N5—C32	−1.3 (2)	C29—C30—C31—C32	−0.4 (4)
O10—C19—C20—C21	−161.7 (2)	C29—C30—C31—C33	−179.0 (3)
O10—C19—C20—C25	18.7 (3)	C30—C31—C32—N5	1.2 (4)
O11—P3—O9—Nd1	142.79 (13)	C30—C31—C32—C36	−178.5 (2)
O11—P3—O12—C27	67.6 (2)	C30—C31—C33—C34	177.8 (3)
O11—P3—N3—C19	−151.4 (2)	C31—C32—C36—N4	−179.6 (2)
O12—P3—O9—Nd1	−104.95 (15)	C31—C32—C36—C35	0.4 (4)
O12—P3—O11—C26	−174.5 (2)	C31—C33—C34—C35	0.7 (5)
O12—P3—N3—C19	101.5 (2)	C32—N5—C28—C29	−0.2 (4)
O13—Nd2—O14—C40	−40.3 (2)	C32—C31—C33—C34	−0.7 (5)
O13—Nd2—O17—P5	−81.78 (15)	C33—C31—C32—N5	179.8 (2)
O13—Nd2—O18—C49	92.6 (3)	C33—C31—C32—C36	0.2 (4)
O13—Nd2—O21—P6	170.12 (13)	C33—C34—C35—C36	−0.1 (5)
O13—Nd2—O22—C58	−165.3 (3)	C33—C34—C35—C37	179.4 (3)
O13—Nd2—N9—C67	111.4 (2)	C34—C35—C36—N4	179.5 (2)
O13—Nd2—N9—C71	−58.71 (19)	C34—C35—C36—C32	−0.5 (4)
O13—Nd2—N10—C75	81.13 (18)	C34—C35—C37—C38	179.3 (3)
O13—Nd2—N10—C78	−87.9 (2)	C35—C37—C38—C39	1.9 (5)
O13—P4—O15—C47	−51.3 (3)	C36—N4—C39—C38	0.3 (4)
O13—P4—O16—C48	157.2 (3)	C36—C35—C37—C38	−1.2 (4)
O13—P4—N6—C40	−33.8 (3)	C37—C35—C36—N4	0.1 (4)
O14—Nd2—O13—P4	2.51 (14)	C37—C35—C36—C32	−179.9 (2)
O14—Nd2—O17—P5	−151.46 (16)	C37—C38—C39—N4	−1.4 (4)
O14—Nd2—O18—C49	39.3 (3)	C39—N4—C36—C32	−179.6 (2)
O14—Nd2—O21—P6	−139.97 (17)	C39—N4—C36—C35	0.4 (4)
O14—Nd2—O22—C58	49.2 (4)	C40—C41—C42—C43	−179.7 (3)
O14—Nd2—N9—C67	166.80 (19)	C40—C41—C46—C45	179.2 (3)
O14—Nd2—N9—C71	−3.3 (2)	C41—C42—C43—C44	0.0 (5)
O14—Nd2—N10—C75	156.68 (18)	C42—C41—C46—C45	−0.2 (5)
O14—Nd2—N10—C78	−12.4 (2)	C42—C43—C44—C45	0.8 (6)
O14—C40—C41—C42	−155.6 (2)	C43—C44—C45—C46	−1.3 (6)
O14—C40—C41—C46	25.0 (4)	C44—C45—C46—C41	1.0 (6)
O15—P4—O13—Nd2	148.39 (14)	C46—C41—C42—C43	−0.3 (4)
O15—P4—O16—C48	−85.8 (3)	C49—C50—C51—C52	179.2 (3)
O15—P4—N6—C40	−159.4 (2)	C49—C50—C55—C54	179.9 (3)
O16—P4—O13—Nd2	−101.05 (16)	C50—C51—C52—C53	1.2 (5)
O16—P4—O15—C47	−166.6 (3)	C51—C50—C55—C54	1.2 (4)
O16—P4—N6—C40	91.7 (2)	C51—C52—C53—C54	0.8 (6)
O17—Nd2—O13—P4	−68.10 (15)	C52—C53—C54—C55	−1.8 (5)
O17—Nd2—O14—C40	65.3 (2)	C53—C54—C55—C50	0.8 (5)
O17—Nd2—O18—C49	−13.6 (3)	C55—C50—C51—C52	−2.2 (4)
O17—Nd2—O21—P6	−68.36 (17)	C58—C59—C60—C61	175.6 (2)
O17—Nd2—O22—C58	103.7 (3)	C58—C59—C64—C63	−177.8 (3)
O17—Nd2—N9—C67	11.9 (3)	C59—C60—C61—C62	2.3 (4)
O17—Nd2—N9—C71	−158.17 (17)	C60—C59—C64—C63	0.0 (4)
O17—Nd2—N10—C75	154.09 (17)	C60—C61—C62—C63	−0.1 (5)
O17—Nd2—N10—C78	−15.0 (3)	C61—C62—C63—C64	−2.1 (5)
O17—P5—O19—C56	−43.0 (3)	C62—C63—C64—C59	2.2 (5)
O17—P5—O20—C57	−54.3 (3)	C64—C59—C60—C61	−2.2 (4)
O17—P5—N7—C49	−20.6 (3)	C67—N9—C71—C70	−1.5 (4)
O18—Nd2—O13—P4	−136.44 (16)	C67—N9—C71—C75	176.7 (2)
O18—Nd2—O14—C40	13.2 (2)	C67—C68—C69—C70	−1.0 (5)
O18—Nd2—O17—P5	−11.98 (14)	C68—C69—C70—C71	−0.6 (5)
O18—Nd2—O21—P6	8.7 (2)	C68—C69—C70—C72	−179.7 (3)
O18—Nd2—O22—C58	178.4 (3)	C69—C70—C71—N9	1.9 (4)
O18—Nd2—N9—C67	36.2 (2)	C69—C70—C71—C75	−176.3 (3)
O18—Nd2—N9—C71	−133.9 (2)	C69—C70—C72—C73	176.7 (3)
O18—Nd2—N10—C75	26.2 (2)	C70—C71—C75—N10	178.5 (2)
O18—Nd2—N10—C78	−142.9 (2)	C70—C71—C75—C74	−1.2 (4)
O18—C49—C50—C51	−161.9 (2)	C70—C72—C73—C74	0.1 (6)
O18—C49—C50—C55	19.5 (3)	C71—N9—C67—C68	−0.1 (4)
O19—P5—O17—Nd2	−96.89 (16)	C71—C70—C72—C73	−2.5 (5)
O19—P5—O20—C57	−173.5 (3)	C72—C70—C71—N9	−178.9 (3)
O19—P5—N7—C49	106.9 (2)	C72—C70—C71—C75	2.9 (4)
O20—P5—O17—Nd2	151.65 (14)	C72—C73—C74—C75	1.6 (5)
O20—P5—O19—C56	74.3 (3)	C72—C73—C74—C76	−177.0 (3)
O20—P5—N7—C49	−146.6 (2)	C73—C74—C75—N10	179.2 (3)
O21—Nd2—O13—P4	54.22 (19)	C73—C74—C75—C71	−1.1 (4)
O21—Nd2—O14—C40	171.5 (2)	C73—C74—C76—C77	179.5 (3)
O21—Nd2—O17—P5	133.12 (14)	C74—C76—C77—C78	0.6 (5)
O21—Nd2—O18—C49	−100.0 (3)	C75—N10—C78—C77	−0.1 (4)
O21—Nd2—O22—C58	−3.8 (3)	C75—C74—C76—C77	0.9 (5)
O21—Nd2—N9—C67	−110.2 (2)	C76—C74—C75—N10	−2.1 (4)
O21—Nd2—N9—C71	79.70 (19)	C76—C74—C75—C71	177.6 (3)
O21—Nd2—N10—C75	−121.53 (19)	C76—C77—C78—N10	−1.1 (5)
O21—Nd2—N10—C78	69.4 (2)	C78—N10—C75—C71	−178.0 (2)
O21—P6—O23—C65	177.9 (3)	C78—N10—C75—C74	1.7 (4)
